# A novel sperm adaptation to evolutionary constraints on reproduction: Pre‐ejaculatory sperm activation in the beach spawning capelin (Osmeridae)

**DOI:** 10.1002/ece3.3783

**Published:** 2018-01-29

**Authors:** José Beirão, Jason A. Lewis, Brendan F. Wringe, Craig F. Purchase

**Affiliations:** ^1^ Department of Biology Memorial University St. John's NL Canada; ^2^ Present address: Faculty of Biosciences and Aquaculture Nord University Bodø Norway; ^3^ Present address: Great Lakes Institute for Environmental Research (GLIER) University of Windsor Windsor ON Canada; ^4^ Present address: Population Ecology Division Bedford Institute of Oceanography, Fisheries and Oceans Canada Dartmouth NS Canada

**Keywords:** adaptation, capelin, evolutionary constraint, fertilization, Osmeridae, sperm biology

## Abstract

Reproduction of external fertilizing vertebrates is typically constrained to either fresh or salt water, not both. For all studied amphibians and fishes, this constraint includes immotile sperm that are activated after ejaculation only by the specific chemistry of the fertilizing medium in which the species evolved (fresh, brackish, or salt water). No amphibians can reproduce in the sea. Although diadromous fishes may migrate between salt and fresh water, they are shackled to their natal environment for spawning in part because of sperm activation. Here, we report for the first time among all documented external fertilizing vertebrates, that in the absence of any external media, sperm are motile at ejaculation in a marine spawning fish (Osmeridae, capelin, *Mallotus villosus*). To illuminate why, we evaluated sperm behavior at different salinities in *M. villosus* as well as the related freshwater spawning anadromous rainbow smelt (*Osmerus mordax*). Surprisingly, sperm performance was superior in fresh water for both species. *M. villosus* spend their entire life at sea but our results show that their sperm are deactivated by sea water, suggesting a freshwater ancestry. By circumventing constraining water chemistry, we interpret the unique pre‐ejaculatory sperm activation in this species as a novel adaptation that enables fertilization in the marine environment. These findings also contribute to understanding the persistence of anadromy, despite great energetic costs to adult fishes.

## 1 INTRODUCTION

In the majority of animal taxa, fertilization and early development are external. In an‐amniotic vertebrates (amphibians and fishes), this constrains each species’ reproduction to one of fresh or salt water, not both, with relatively few reproducing at intermediate conditions (e.g., Tiersch and Yang 2012). In addition to embryonic homeostasis, this physiological constraint includes the ability of sperm to swim (Browne et al., 2015; Reinhardt, Dobler, & Abbott, 2015). Sperm of externally fertilizing amphibians and fishes are immotile within seminal plasma ~10psu (osmolality ranging from 200 to 400 mOsmol/kg (Alavi & Cosson, 2006; Cosson, 2010)). At ejaculation, sperm motility activates instantly on contact with an appropriate external medium (plain fresh or salt water in most documented cases). A substantial chemical change in the fertilizing environment requires either (i) post‐ejaculated sperm swimming performance to evolve via natural selection so that populations have sperm locally adapted to their environments (this has been demonstrated on a fine scale in the frog *Crinia signifera* (Byrne, Dunne, Munn, & Silla, 2015), and the sand goby *Pomatoschistus minutus* (Svensson et al., 2017)), or (ii) individuals to migrate for reproduction. Although diadromous fishes migrate across marine and freshwater habitats (catadromous salt water spawn, fresh water growth; anadromous fresh water spawn, salt water growth), they must return to their natal environment for spawning because typically marine fish sperm activate upon hypertonic shock with sea water (no activation in fresh water), whereas freshwater fish (and amphibian) sperm activate upon hypotonic shock or decrease in specific ions (Alavi & Cosson, 2006; Browne et al., 2015; Cosson, 2010).

An‐amniotic vertebrates are therefore tied to fresh or marine spawning based on their evolutionary history. No amphibians can reproduce in salt water. Very few fish species can activate sperm in both marine and freshwater environments (published exceptions are via acclimation processes in killifish, stickleback, and tilapia ((Tiersch & Yang, 2012; Legendre et al., 2016) and references within (Taugbøl, Mazzarella, Cramer, & Laskemoen, 2017)) or specialized female secretions in a stickleback (Elofsson, McAllister, Kime, Mayer, & Borg, 2003; Elofsson, Van Look, Sundell, Sundh, & Borg, 2006)). Comparative studies on the reproductive physiology of species from families containing marine and freshwater spawners may provide important insights into this constraint.

**Figure 1:**
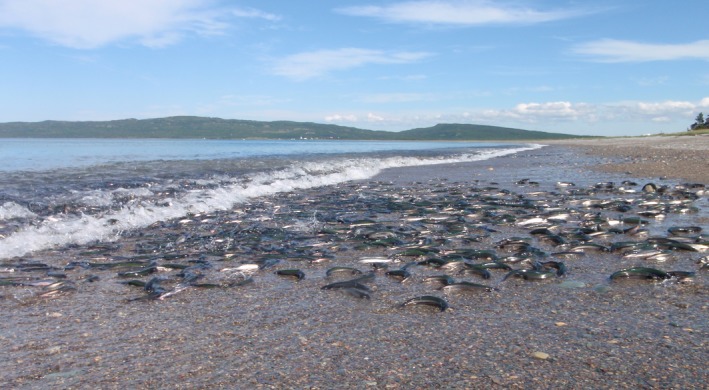
Capelin (*Mallotus villosus*) spawning on a Newfoundland beach. Beach spawning *M. villosus* gather close to the shore and then vigorously roll together in pairs or trios (two males and one female in the center) into the beach against the receding wave. Fertilization occurs in the few seconds between breaking waves and eggs immediately adhere to the sediment (see Templeman, 1948 for a detailed description).

The northern hemisphere smelts of the Family Osmeridae are mostly freshwater spawners (Table 1). However, some have unusual reproductive habits, such as marine beach spawning at varying levels of salinity in capelin (*Mallotus villosus*) (Figure 1), surf smelt (*Hypomesus pretiosus*), Japanese smelt (*H. japonicas*), and night smelt (*Spirinchus starksi*). The family's evolutionary origin (Beirão, Lewis, & Purchase, 2015; Dodson, Laroche, & Lecomte, 2009; Ilves & Taylor, 2009) and whether spawning evolved from marine to fresh water or vice versa (Dodson et al., 2009; Gross, Coleman, & McDowall, 1988) is debated. This study is the first work on sperm behavior of a marine spawning member of this family. Here, we report an observation that left us astonished, that *M. villosus* sperm are motile upon leaving the body and are quickly immobilized by sea water (their native habitat). This has not been reported in any other vertebrate with external fertilization. Based on the uncertainty of the evolutionary origins of the Osmeridae, we build the hypothesis that *M. villosus* pre‐ejaculatory sperm activation is an alternative route to circumvent evolutionary constraints of water chemistry. To help support this hypothesis, we measured phenotypic plasticity in sperm behavior to different salinities in the marine spawning *M. villosus* and in the related anadromous (i.e., freshwater spawning) rainbow smelt *Osmerus mordax*. We found sperm performance to be superior in fresh water for both species. *M. villosus* exhibit unique sperm behavior for any an‐amniotic vertebrate, an adaptation which would enable a freshwater‐derived taxon to spawn in the sea.

**Table 1:** Spawning locations of the Osmeridae (Martin, 2015; Martin & Swiderski, 2001; McAllister, 1963, www.fishbase.org), and what is known of their sperm biology (most species have no published information)

Species (common name)	Spawning location	Sperm activation
*Osmerus eperlanus* (European smelt)	Rivers	‐Immotile at stripping ‐Motility inhibited in the presence of 100 mM K^+^ ‐Higher activation in 20 mM NaHCO_3_ and 120 mMNaCl (Kowalski et al., 2006)
*Osmerus mordax* (rainbow smelt)	Rivers	‐Immotile at stripping ‐Highest motility between 0 and 10psu ‐Immotile in water >15psu (DeGraaf & Berlinsky, 2004; and this study)
*Osmerus dentex* (Arctic rainbow smelt)	Rivers	
*Osmerus spectrum* (pigmy smelt)	Rivers	
*Thaleichthys pacificus* (eulachon)	Rivers	
*Hypomesus olidus* (pond smelt)	Rivers	
*Hypomesus nipponensis* (wakasagi)	Rivers	
*Hypomesus transpacificus* (delta smelt)	Rivers	
*Hypomesus pretiosus* (surf smelt)	**Surf (beaches)**	
*Hypomesus japonicas* (Japanese smelt)	**Beaches**	
*Spirinchus thaleichthys* (longfin smelt)	Rivers	
*Sprinchus lanceolatus* (shishamo)	Rivers	‐Immotile at stripping ‐Motility duration decreases >20 mM K^+^, some motility to 200 mM K^+^ ‐Highest activation in 100 mOsm NaCl (Ohta et al., 1995)
*Spirinchus starksi* (night smelt)	**Surf (beaches), with fresh water incursion**	
*Allosmerus elongates* (whitebait smelt)	**“Probably in ocean,” subtidal banks**	
*Mallotus villosus* (capelin)	**Beaches, inshore subtidal, offshore**	‐Motile at stripping ‐Highest motility between 5 and 10psu ‐Very low motility >20psu (this study)

Those in **bold** are reported to spawn in contact with some salinity range of salt water.

## 2 MATERIAL AND METHODS

### 2.1 *Mallotus villosus* initial sperm observations

There are no published reports of sperm behavior in this species. In 2010, spawning *M. villosus* were captured (>1000 individuals, with microscopic observations on >50 male's sperm) on Bellevue Beach (47^o^64′N, 53^o^78′W), from the Avalon Peninsula, Newfoundland, Canada. The typical approach of diluting semen with water collected from spawning locations resulted in no motile sperm being observed. Contrary to what would be expected for any external fertilizing vertebrate we discovered that *M. villosus* sperm are motile upon leaving the body and are quickly immobilized by sea water. Subsequent work across multiple years (2011 – 2017) with hundreds of fish from different spawning locations along the coast of Newfoundland has corroborated the ubiquity of this observation. These observations led to the experiment reported here. In the related freshwater spawning *O. mordax*, as in other externally fertilizing vertebrates, sperm are immotile upon leaving the body and are activated upon contact with water.

### 2.2 Experimental fish origin and semen collection

Anadromous *O. mordax* make daily nocturnal migrations into fresh water to spawn just upstream of high tide. Spawning *O. mordax* were captured in the Salmonier River (47^o^19′N, 53^o^39′W) and spawning *M. villosus* on Bellevue Beach (47^o^64′N, 53^o^78′W), both from the Avalon Peninsula, Newfoundland, Canada. Fish were transported to Memorial University and kept for a maximum of 2 days in 7–10°C flow‐through aerated seawater tanks until sampled. Ten of each species were sequentially killed (overdose of MS‐222), quickly patted dry, and using a pipette their semen collected from the genital duct by squeezing the abdomen. All procedures followed Canadian guidelines on the use of research animals (Memorial University protocol 12‐07‐CP).

### 2.3 Sperm activation

Immediately after collection, semen was diluted in a non‐activating solution and kept at 5°C until analyzed (within 1 hr). This solution was developed based on artificial seminal plasma used in a related species (Ohta, Unuma, Tsuji, Yoshioka, & Kashiwagi, 2001): 130 mM KCL, 0.6 mM CaCl_2_, 2.3 mM MgCl_2_, 20 mM NaHCO_3_, 20 mM HEPES (pH 7.0‐7.5) with 0.1% BSA to prevent sperm cells from adhering to microscope slides. Solutions without K^+^ failed to deactivate *M. villosus* sperm. Different dilutions, 1:10 and 1:50, were used for *O. mordax* and *M. villosus,* respectively. These were the lowest semen:diluent ratios that prevented sperm motility in each species. *M. villosus* spawned 5 weeks after *O. mordax* and we discovered the 1:10 dilution was not adequate for *M. villosus* sperm. Later experiments (subsequent year) with *O. mordax* sperm confirmed that 1:10 and 1:50 dilutions give similar post‐activation sperm swimming results (unpublished data).

Diluted sperm were re‐activated to examine phenotypic plasticity in sperm performance (Purchase, Butts, Alonso‐Fernandez, & Trippel, 2010) to environmental salinity using seven solutions (0, 5, 10, 15, 20, 25, and 30psu, with 0.1% BSA) that were prepared by adding Instant Ocean^®^ sea salt to distilled water. All salinities were examined in detailed for 10 *M. villosus*. For *O. mordax*, due to a logistical problem, all seven salinities were tested using five individuals, while only 0 and 10psu water was tested on the other five animals.

The microscope plate and glass slide (2X‐CEL Dual Sided Sperm Analysis Chamber, Hamilton Thorne Biosciences) were pre‐chilled to 8°C with a customized Physitemp TS‐4 system. Diluted semen (0.1 μl) was added to a single well of the two‐welled slides, and sperm were immediately activated by adding 1 μl (*O. mordax*) or 1.5 μl (*M. villosus*) test water at 5°C. Videos were acquired with an inverted Leica DM IL LED microscope (Leica Microsystems, Concord, ON, Canada), using a 20 ×  phase contrast objective, and a Prosilica GE680 monochrome camera (Allied Vision Technologies, Burnaby, BC, Canada) set at 100 frames per second. Videos were analyzed with a computer assisted sperm analysis (CASA) plugin for ImageJ (Purchase & Earle, 2012; Wilson‐Leedy & Ingermann, 2007). Input parameters were chosen so the software could differentiate drifting from motile sperm and are described in Table S1. The software determined the percent of the sperm cells that were motile (motility, MOT), and for motile cells, the curvilinear swimming velocity (VCL), which was strongly correlated (Pearson's correlations, *r* at least .74, *n* = 627, *p* < .0001) with the velocity along a derived smoothed path (VAP) and straight line velocity (VSL = displacement). To simplify analyses, we chose to proceed with MOT and VCL as measures of sperm performance (Purchase & Moreau, 2012). We selected VCL from the different velocities, as it represents the actual sperm velocity. Most fertilizations likely occur very quickly, and it took less than 30s for virtually all moving sperm to become non‐motile (Figure S1); therefore, we chose to use 5s after sperm:water mixing to evaluate sperm behavior at different salinities (the earliest time that was possible to capture a high quality CASA video). Two subsamples of each animal's semen were recorded at each salinity for technical replication. These were averaged, and means of these subsamples are presented among individuals.

## 3 RESULTS

Between 2010 and 2012, before the main experiment, several collections from different spawning locations along the coast of Newfoundland with hundreds of different fish led to the observation that *M. villosus* sperm is motile upon leaving the body. In all instances when sea water collected from the spawning locations was added to swimming sperm the motility rapidly ceased. Because of this situation, the semen could not be diluted and the sperm activated by water at the same time, as is typical of CASA work. Therefore, we had to develop a solution to de‐activate sperm motility before semen could be mixed with water.

**Figure 2:**
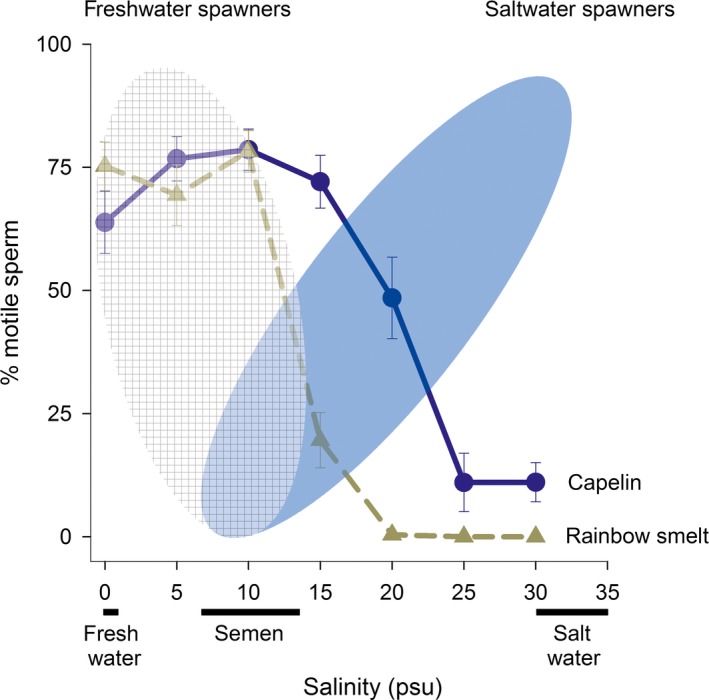
Sperm motility re‐activation in *Osmerus mordax* (brown dashed line) and *Mallotus villosus* (blue solid line) in response to salinity. Data are means ± *SE* among 10 animals 5s after sperm exposure to water, from our experiment. Sperm of externally fertilizing an‐amniotic vertebrates are typically immotile at the osmolality of semen, which varies across species (~200–400 mOsmol/kg). The exact shape of the response curve is species dependent (cartoon ellipses are drawn to show general patterns; (Alavi & Cosson, 2006; Browne et al., 2015)), but sperm motility is usually activated by hypertonic (blue solid ellipse = marine spawners, e.g., cods) or hypotonic (brown checker ellipse = freshwater spawners, e.g., carps) shock upon ejaculation.

**Figure 3:**
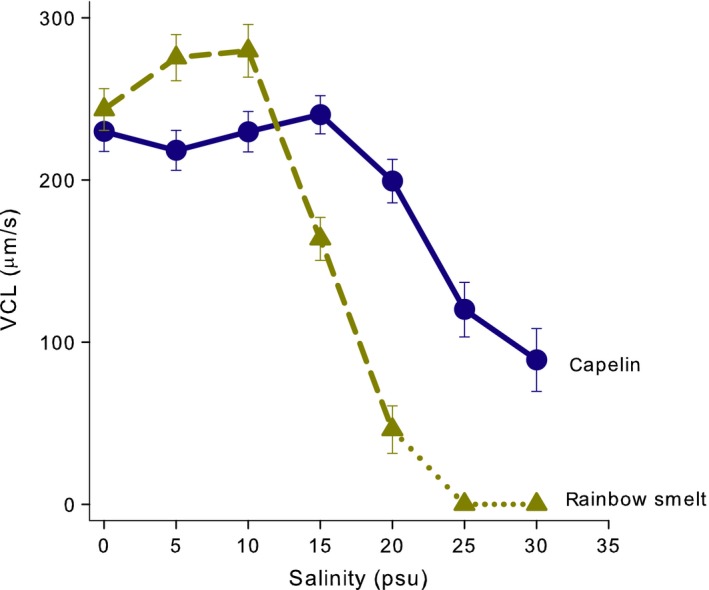
Curvilinear swimming velocities (VCL) of motile sperm cells of *Osmerus mordax* (brown dashed line) and *Mallotus villosus* (blue solid line) in response to salinity, from our experiment. Data are means ± *SE* among 10 animals 5s after sperm exposure to water. Only 0.4% of *O. mordax* sperm were motile at 20psu and none at higher salinities (conceptual reaction norm is drawn as dotted line).

When tested at conditions typical of where the species spawns (≥30psu), artificially immobilized *M. villosus* sperm had poor re‐activation (Figure 2) and swimming velocity was low (Figure 3). However, *M. villosus* sperm were readily re‐activated by fresh water, with % motility being highest in the range of 5–10psu, after which there was a decrease, while very few cells activated in water with salinity above 20psu (Figure 2). Similarly, sperm swimming velocities were highest at low salinities, with a drop at 25psu (Figure 3). For context, the typical relationship between salinity and motility for freshwater and marine spawners is illustrated in Figure 2.

As expected for a freshwater spawner, sperm activation and swimming velocity in *O. mordax* were greatest at low salinity. Motility dropped above 10psu; only 0.4% of sperm showed movement at 20psu and none did at higher salinities (Figure 2). Sperm swimming velocities were highest at 5–10psu, with a substantial decrease in the velocity at 15psu. For both species, initial velocities were high at 5s (Figure 3) but few cells maintained motility after 15s regardless of salinity (Figure S1).

## 4 DISCUSSION

Most Osmeridae spawn in fresh water (Dodson et al., 2009; McAllister, 1963), and previously studied (Table 1) osmerids (*O. mordax*,* O. eperlanus,* and *S. lanceolatus*) show predictable sperm responses to such conditions (DeGraaf & Berlinsky, 2004; Kowalski et al., 2006; Ohta, Kusuda, & Kudo, 1995). *O. mordax* exist either as freshwater residents or anadromous populations that return to fresh water to spawn. Our observation that their sperm are incapable of activation in salt water likely contributes to the constraint of anadromous individuals spawning in fresh water, similar to salmonids. Freshwater spawning osmerid sperm is not activated by hypotonic shock (as in cyprinids), but like in salmonids by a decrease in K^+^, as was demonstrated in *O. eperlanus* (Kowalski et al., 2006).

This is the first study to report an external fertilizing vertebrate (*M. villosus*) with sperm that leave the body already activated. The result of superior sperm performance in fresh water for both freshwater spawning *O. mordax* and marine spawning *M. villosus* is more indicative of freshwater than marine fishes (Alavi & Cosson, 2006; Cosson, 2010). Our sperm research suggests a freshwater rather than marine ancestry for these species, as traditionally supported (e.g., McDowall, 2002). Nonetheless, more recently Dodson et al. (2009), based on a phylogenetic analysis of the suborder Osmeroidei (Osmeriformes), predicted that some anadromous species evolved from marine spawners. They conclude that freshwater spawning osmerids are most likely derived from a marine ancestor that exploited low salinity to reduce mortality on offspring. Our results are in conflict with such a conclusion. Thus, our results add important information to the debate in the literature about the origin of anadromy (Dodson et al., 2009; Gross et al., 1988) a topic that deserves further study. Sperm characteristics of the other marine spawning osmerid species should be investigated.

Based on their sperm performance, successful *M. villosus* reproduction in the marine environment is perplexing. The fertilization environment of beach spawning *M. villosus* in Newfoundland varies substantially on fine temporal and spatial scales but usually occurs at salinities higher than 30psu. This salinity quickly immobilizes their sperm and poorly re‐activated the artificially immobilized sperm in our experiment. We believe reproduction is possible through the combination of at least four mechanisms. First, at times salinities on and within the beach could be lower than coastal sea water (Prӕbel, Christiansen, & Fevolden, 2009), and our work indicates this would prolong sperm motility and increase sperm swimming velocity. Secondly, ovarian fluid, which is released with eggs and influences sperm in other species (e.g., Beirão, Purchase, Wringe, & Fleming, 2015; Elofsson et al., 2006), may improve sperm performance of *M. villosus* at higher salinities. However, ongoing research at our laboratory indicates that only ~1% of the egg volume in *M. villosus* is ovarian fluid (unpublished data). *M. villosus* spawn as pairs/trios in very tight contact in the intertidal zone, in between breaking waves (Templeman, 1948) and eggs must be fertilized and adhered to the substrate in the 2‐3 seconds before the next wave washes the gametes away. As a result, the sperm may always swim in a mixture of ovarian fluid and water (never full strength salinity). Thirdly, swimming velocity of *M. villosus* sperm is high compared with most other fishes (Cosson, 2010), helping in rapid fertilization before sperm are immobilized by sea water on the next breaking wave. Finally, and most importantly, we interpret pre‐ejaculatory sperm activation in *M. villosus*, reported in this work, as a unique adaptation, freeing them of the need for an appropriate external medium for sperm activation and enabling reproduction in the sea. The exact mechanism of sperm pre‐activation is currently being researched in our laboratory. Other examples of vertebrates with sperm that leave the body already motile are internal fertilizers; sperm behavior of fishes with internal fertilization (e.g., Yao, Crim, Richardson, & Emerson, 2000) being more similar to that of amniotic tetrapods (Birkhead, Hosken, & Pitnick, 2009).

Around the island of Newfoundland *M. villosus* spawn predominantly on beaches, but also on the seafloor in nearshore areas (Nakashima & Taggart, 2002). These groups are not genetically distinct populations (Penton, McFarlane, Spice, Docker, & Davoren, 2014), but rather spawning choices attributed to temperature by some authors (Davoren, 2013). Our results add to the difficulties in reproductive success faced by subtidal spawning *M. villosus* as predicted by Nakashima and Wheeler (2002). In the North Atlantic, other populations of *M. villosus* spawn offshore on the continental shelves (Carscadden, Frank, & Miller, 1989; Davenport, 1989; Prӕbel, Christiansen, Kettunen‐Prӕbel, & Fevolden, 2013), but beach spawning is believed to be the ancestral condition (Martin, 2015; Purchase, 2018; Vilhjálmsson, 1994). Given offshore spawning is likely the derived state, we predict their sperm are more tolerant of sea water than those of beach spawning populations. Whether those spawning on nearshore seafloors have salinity tolerance closer to beach or offshore populations is unknown (Purchase, 2018).

Martin and Swiderski (2001) concluded anadromy led to marine beach spawning in *M. villosus* and two other osmerids (*H. pretiosus* and *S. starski*), an opinion supported by our data. These observations are paralleled by Purchase (2018), which reports that in vitro fertilized eggs from beach spawning *M. villosus* have good hatch success when incubated in 2–28psu, but it is poor at higher salinities. The benefits of changing from stream to beach spawning are not obvious, as there are several risks (Martin, 2015). Spawning habitat availability could have played an important role in this habitat transition (Martin & Swiderski, 2001). In general, our observations contribute to the understanding of the persistence of anadromy despite the energetic costs associated with the migrations between the fresh water and marine environment (e.g., Dodson et al., 2009).

## CONFLICT OF INTEREST

None declare.

## AUTHOR CONTRIBUTIONS

JB co‐designed the study, performed the experiments, and wrote the initial draft of the manuscript. JL performed the experiments. BW participated in preliminary experiments and manuscript writing. CP conceived, co‐designed, and coordinated the study and finalized the manuscript.
