# Supplementary material for: A novel sperm adaptation to evolutionary constraints on reproduction: Pre‐ejaculatory sperm activation in the beach spawning capelin (Osmeridae)
Source: Ecol Evol. 2018 Jan 29;8(4):2343–9. doi: 10.1002/ece3.3783 (PMC5817138; doi:10.1002/ece3.3783)
Supplement: Supplementary file 2 [file ECE3-8-2343-s002.doc]

**Supplementary material**

Table S1: Parameter input used to set the ImageJ CASA plugin.

| **Parameter** | **Value** |
| --- | --- |
| (a) Minimum sperm size (pixels) | 2 |
| (b) Maximum sperm size (pixels) | 7 |
| (c) Minimum track length (frames) | 50 |
| (d) Maximum sperm velocity between frames (pixels) | 5 |
| (e) Minimum VSL for mobile (μm/s) | 5 |
| (f) Minimum VAP for mobile (μm/s) | 20 |
| (g) Minimum VCL for mobile (μm/s) | 25 |
| (h) Low VAP speed (μm/s) | 5 |
| (i) Maximum percentage of path with zero VAP | 1 |
| (j) Maximum percentage of path with low VAP | 25 |
| (k) Low VAP speed 2 (μm/s) | 20 |
| (l) Low VCL speed (μm/s) | 60 |
| (m) High WOB (percent VAP/VCL) | 80 |
| (n) High LIN (percent VSL/VAP) | 80 |
| (o) High WOB two (percent μm/s) | 50 |
| (p) High LIN two (percent μm/s) | 60 |
| (q) Frame Rate (frames/s) | 100 |
| (r) Microns per 1000 pixels | 956 |
